# A scoping review exploring carbon emissions in dentistry—a step towards sustainability

**DOI:** 10.1186/s12903-025-06952-w

**Published:** 2025-10-27

**Authors:** Rakshita Chalotra, Ramya Shenoy, Parul Dasson Bajaj, Ashwini Rao, Mithun Pai, Praveen Jodalli, Avinash BR, Harsh Priya, Violet D’Souza

**Affiliations:** 1 Department of Public Health Dentistry, Manipal College of Dental Sciences Mangalore, Manipal Academy of Higher Education, Manipal, India; 2 Department of Public Health Dentistry, CDER, AIIMS, New Delhi, India; 3 Dept. of Dental Clinical Sciences, Faculty of Dentistry, Dalhousie University, Halifax, Canada

**Keywords:** Carbon footprint, Sustainable development, Dental care, Dental waste, Climate change

## Abstract

**Background:**

In the context of advancing sustainability in healthcare, in alignment with Sustainable Development Goal 13 on Climate Action, there is a necessity to enhance our understanding of the carbon footprint associated with dental services to establish an environmentally conscious system. This insight could help devise strategies for policymakers, oral health practitioners, and researchers to establish more sustainable dental practices. Therefore, the present scoping review was conducted to analyse the current body of literature exploring carbon emissions generated from dental health care services with the aim of promoting sustainable dental practices.

**Methods:**

This scoping review was conducted in line with Arksey and O’Malley’s Framework and was reported using the PRISMA-ScR guidelines. A systematic database search was conducted across four databases: PubMed, Scopus, Web of Science, and EMBASE, to collect original research published in English up to June 2024, focusing on carbon emissions in dentistry. Review articles, letters to editors, and editorials were excluded. Selected articles underwent critical appraisal using Crowe’s Critical Appraisal Tool, followed by data charting and thematic analysis using Atlas.ti for Mac version 24.1.0.

**Results:**

A total of 15 articles were included in this review based on the eligibility criteria. The qualitative thematic analysis of these articles revealed four main themes: sources of carbon emissions, strategies to promote sustainable dental practices, challenges for sustainability, and future research areas.

**Conclusions:**

The review provides a comprehensive understanding of the various sources of carbon emissions associated with dental practices along with the challenges faced towards sustainable clinical practices. The key themes were explored across four hierarchical levels: structural, dental practice, dental practitioner, and method or product. The findings further guided us toward potential strategies for integrating sustainability into dental care, including stakeholder collaboration, policy reform, management practices, infrastructural improvements, and the adoption of environmentally-friendly materials. Additionally, the review highlights the need for future research to explore sustainable alternatives, conduct life cycle impact assessments, and undertake more qualitative studies to inform best practices.

**Supplementary Information:**

The online version contains supplementary material available at 10.1186/s12903-025-06952-w.

## Background

The global imperative to address climate change has placed sustainability at the forefront of policy and practice across all sectors, including healthcare [1, 2]. According to the International Organization for Standardization (ISO), sustainability is the condition where present needs are met without compromising future generations and any practice or product which supports or maintains this balance across environmental, social, and economic systems is considered sustainable [3]. In alignment with this principle, reducing carbon emissions remains a global priority, with several nations taking action through initiatives such as United Kingdom’s Climate Change Act of 2008, the Paris Agreement of 2016 and also New Zealand’s Zero Carbon Act of 2019, which aims for carbon neutrality [1, 4].

Healthcare systems are significant contributors to carbon footprint, accounting for approximately 4.4% of global net emissions [5]. Additionally, the World Health Organization (WHO) acknowledges the environmental effects of health systems and their potential to significantly reduce carbon emissions, thereby fostering a sustainable healthcare system. Sustainability in healthcare emphasizes improving processes through efficient resource management and strategic planning, reducing waste, conserving energy, optimizing procurement, and redesigning care delivery processes to minimize environmental harm while maintaining high-quality patient outcomes [6].

Dentistry, as a specialized sector within healthcare, plays a critical yet often underrecognized role in environmental sustainability. Within the field of dentistry, the gravity of the problem can be simply understood by considering the carbon emission from a single Root canal treatment (RCT) i.e. 4.9 kg CO2eq which is comparable to driving a small car for 30 km [7]. Dental practices contribute to carbon emissions through multiple pathways including travel, energy use, materials procurement, and biomedical waste [8]. Even though a single dentist produces very little biomedical waste, the cumulative waste has a big influence on the environment [9]. Dental healthcare’s extensive reliance on both disposable and reusable products highlights the need to transcend the concept of sustainability beyond mere carbon emissions, to encompass the choice of materials procured, the waste generated, and the broader impact on biodiversity [10–12].

Therefore, the concept of sustainability in dentistry should focus on delivering high-quality oral healthcare while emphasizing preventive practices, responsible material procurement, conservation of resources and energy, and efficient waste management—all contributing to environmentally friendly operations [13]. The term “environmentally friendly,” as defined by ISO, refers to products and practices that cause reduced, minimal, or no environmental harm, and will be used throughout this review to refer to such practices or products [14]. Among the examples of environmentally friendly dental practices, Taiwan is contributing by integrating sustainability into practice with efforts ranging from a decline in dental amalgam use to the adoption of digital imaging, electronic record keeping and intraoral scanners, thereby reducing paper, plaster, and chemical waste [15–17]. These sustainable practices extend into dental education through the use of haptic virtual reality dental simulators for pre-clinical training for hand skills thereby moving towards greener training alternatives by minimizing the need for synthetic materials [18].

While such efforts illustrate meaningful progress in advancing towards sustainability in dentistry, the broader evidence base remains fragmented. Although studies have addressed individual sources of emissions or specific interventions, there is limited synthesized knowledge that integrates these findings into a comprehensive understanding of the field. A clearer understanding of how different dental procedures contribute to carbon emissions, along with the strategies to reduce them, is crucial for shaping clinical guidelines and informing future research and policy decisions [19]. Therefore, the aim of this scoping review was to examine and synthesize the current body of literature on carbon emissions associated with dental healthcare services, particularly in light of the shift towards sustainable dental practices. In doing so, this review supports ongoing efforts to align dental care with the broader goals of environmental sustainability and global climate action, particularly those outlined in Sustainable Development Goal 13 [20].

## Methods

This scoping review has been reported in alignment with PRISMA-ScR guidelines [21] with the checklist provided as supplementary file 1 and the review protocol was not registered prior to the review process. This review was conducted using Arksey and O’Malley’s framework [22], with a detailed description of the first five stages provided in the following sections. The optional sixth stage, stakeholder consultation, was not carried out for this review due to time and resource constraints.

### Identifying the research question

The present scoping review was conducted to answer the research question: “What is the current body of literature exploring carbon emissions generated from dental health care services with the aim of promoting sustainable dental practices?” To enhance clarity, the research question is further delineated using the PCC (Population-Concept-Context) framework. The Population comprised dental health care services and providers involved in oral health care delivery. The Concept focused on carbon emissions associated with dental practices and related strategies to enhance sustainability in clinical practice while the Context encompassed global healthcare settings where dental services are provided, with an emphasis on sustainable practice implementation and policy relevance.

### Identifying relevant literature

A systematic search of literature was conducted across four databases i.e., PubMed, Scopus, Web of Science and EMBASE by one author (RC) using the following keywords: ‘dental’, ‘dental care’, ‘dental waste’, ‘carbon’, ‘carbon footprint’, ‘emission’, ‘hospital’, ‘carbon emission’ and ‘hospital waste’. The search strategy was solely developed by the authors and customized across all databases as listed in Supplementary File 2, with the most recent search conducted on 30th June 2024. No date restrictions were applied; however, the search was limited to articles published in English. Additionally, backward citation searching was carried out from the selected articles to identify other potential studies.

### Study selection

This scoping review included original research which explored carbon emissions in dentistry to move towards sustainable dental practice. Additionally, this review included articles published in English up to 30 June, 2024. Articles with missing full-texts, review articles, editorials and letter to editors were excluded from this scoping review. The articles identified following initial search were managed using Mendeley Reference Manager [23] and duplicates were removed using Rayyan.ai [24]. Based on the eligibility criteria, the articles which were identified after initial title screening by one author (RC) underwent title and abstract screening by two authors (RC and RS) followed by full-text screening. Any disagreements at this stage were resolved through discussion with third author (PDB), and consensus was subsequently reached.

### Charting the data

Two authors (RC and RS) independently extracted data pertaining to the research question from the selected full-text articles using a predetermined framework. This included details such as the study’s title, authors, year of publication, theoretical and conceptual orientation purpose, research questions and/or hypothesis, sample description, methods and analysis, key measures, main findings or results, study limitations mentioned by the author(s), study strength and importance were all compiled. The data extraction tables were collectively reviewed to identify and complete any missing information relevant to the review.

### Collating, summarizing and reporting the results

Although an optional step in scoping review methodology, the quality appraisal, was undertaken to systematically identify existing research gaps, critically evaluate the methodological rigor of current studies, and guiding future research directions in this field. The Crowe Critical Appraisal Tool (CCAT) was employed to assess the quality of the sources of evidence for the present scoping review [25]. CCAT involves evaluating each article across eight categories, with scores ranging from 0 to 5 for each category, and a maximum score of 40 for each article. Based on the CCAT scores, the articles were scored as high quality with scores more than 35, medium quality with scores between 25 and 34 and low quality for scores below 25 [26]. Following the completion of the critical appraisal the first author (RC), a second author (RS) independently reviewed a randomly selected subset of 8 out of the 15 included articles to assess scoring consistency and ensure reliability. This process yielded an intraclass correlation coefficient (ICC) of 0.99, demonstrating a high level of agreement between the authors.

In addition to data extraction from the selected studies, two independent researchers (RS and PDB) conducted qualitative thematic analysis using ATLAS.ti 24.1.0 for Mac [27]. To ensure familiarity with the data, both researchers independently read all articles multiple times and reviewed them in conjunction with the data extraction table. Following this, important segments in the articles relevant to carbon emissions in dentistry and sustainability practices were independently highlighted and initial codes were assigned to these segments using an inductive approach.

Subsequently, the researchers collaboratively reviewed and compared their initial codes, engaging in multiple rounds of discussions to resolve any discrepancies and merge redundant codes, ultimately arriving at the final codebook through this iterative coding process. The final codes were further examined to identify patterns and similarities, which were then organized into broader categories. Through ongoing discussion and comparison, these categories were then refined and grouped into sub-themes and main themes to effectively capture the underlying meanings and relationships within the data. The resulting thematic structure has been tabulated to enhance transparency and provide a clear overview of the coding framework, thereby adequately addressing the research question.

## Results

**Fig. 1:**
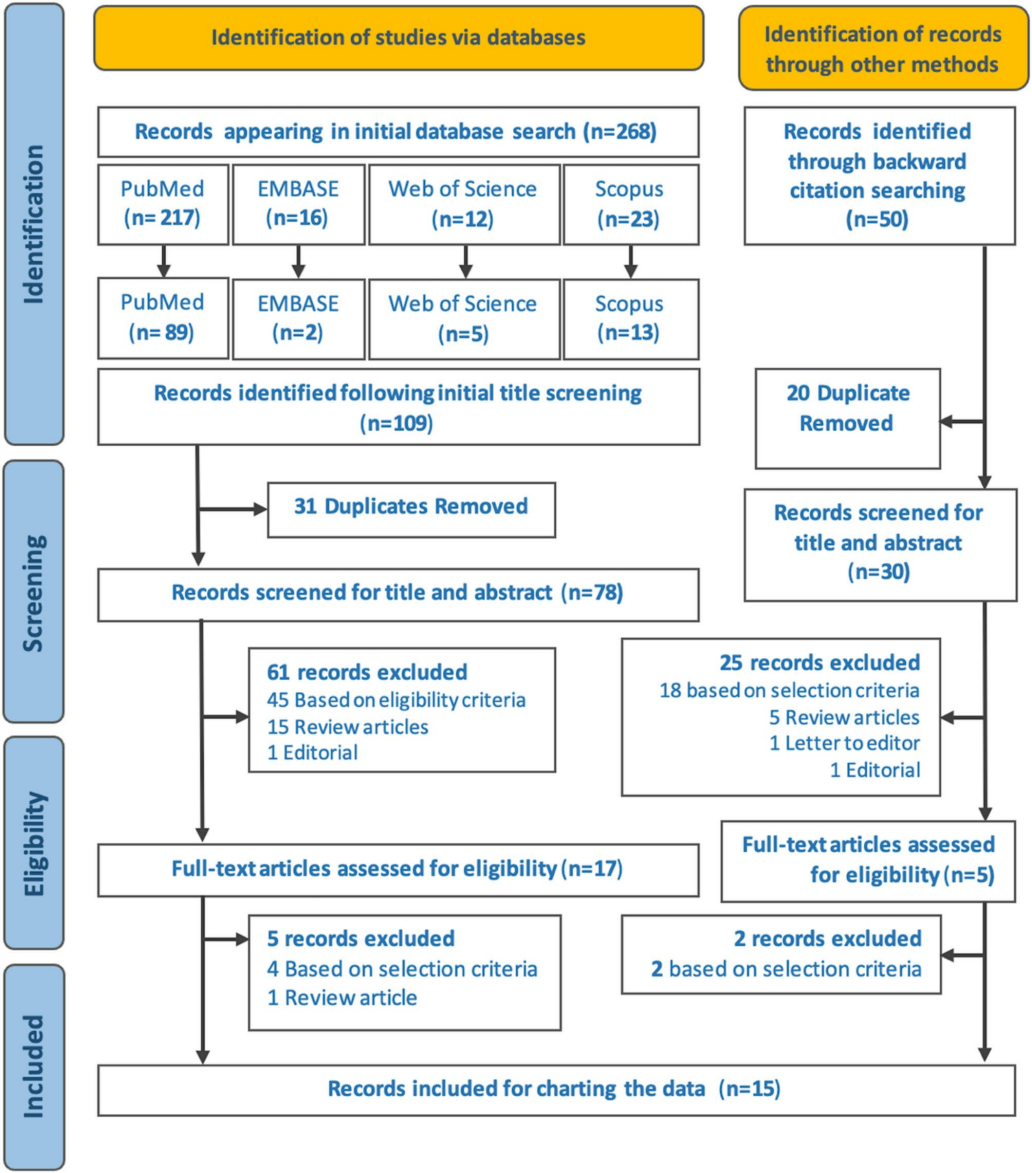
Prisma flow diagram depicting the study selection process

A total of 268 records were found in the initial search across the four databases, leading to the identification of 109 articles after the initial title screening. After removing duplicates, 78 articles underwent title and abstract screening. Subsequently, 17 articles were reviewed in full-text, and 12 articles were selected. Following this, a backward citation search was performed on these selected articles, resulting in the addition of 3 more articles to the total pool of 15 articles selected for this scoping review. The study selection process is illustrated using the PRISMA flow diagram [28] [Fig. 1], while Supplementary File 3 provides detailed information on both the included and excluded studies.

Among the sources of evidence included in this scoping review, the publication timeline ranged from 2012 to 2024. In terms of geographical distribution the majority of studies were conducted in the United Kingdom [1, 2, 9, 19, 29–32] with a few based in Sweden [7, 33], Ireland [34, 35], United States of America [36] and Netherlands [37]. Of the 15 studies included, 7 were life cycle analysis [7, 9, 30, 31, 33–35], 3 were quantitative studies [8, 19, 32], 2 were qualitative studies [29, 37], 2 were waste audits [2, 36] and 1 employed a mixed methods design [1]. Several studies focused on specific dental procedures, including one each on root canal treatment [7], dental examination [33], nitrous oxide sedation [31], fluoride varnish application [34], toothbrush use [19] and preclinical training exercises [36]. Notably, three studies were conducted in dental practices that served both National Health Service (NHS) and private patients [1, 2, 29]. A detailed summary of the characteristics of the sources of evidence is provided in the data extraction table available as Supplementary File 4.

Based on the CCAT scores, four studies were classified as high quality, while the majority of the studies were assessed to be of medium quality, reflecting moderate methodological rigor most likely linked to reporting clarity. In contrast, one study was rated as low quality due to weaknesses in study design, limited transparency in methodology and insufficient justification of results. Table 1 presents a comprehensive breakdown of individual CCAT scores for each study.

**Table 1:** Crowe’s critical appraisal scores of the included studies

Author and year of publication	Preliminaries	Introduction	Design	Sampling	Data Collection	Ethical	Results	Discussion	Total	Quality of the study
Duane et al., 2012 [8]	3	3	3	4	3	2	4	4	26	Medium
Grose et al., 2016 [29]	5	5	4	3	3	4	4	5	33	Medium
Richardson et al., 2016 [2]	4	5	4	4	5	5	5	5	37	High
Duane et al., 2017 [19]	4	3	3	3	3	1	4	4	25	Medium
Grose et al., 2018 [1]	4	4	3	3	3	4	4	3	28	Medium
Duane et al., 2020 [7]	5	5	4	4	4	5	5	5	37	High
Lyne et al. 2020 [9]	4	4	4	4	3	3	3	4	29	Medium
Borglin et al. 2021 [30]	5	5	4	4	4	4	5	5	36	High
Lyne et al. 2022 [31]	5	5	4	3	4	4	4	5	34	Medium
Volgenant et al. 2022 [32]	4	3	4	4	4	5	4	4	32	Medium
Duane & Steinbach 2024 [33]	5	5	4	3	3	5	4	4	33	Medium
Fennell-Wells et al. 2024 [34]	5	5	5	4	5	4	5	5	38	High
Martin et al. 2024 [35]	4	5	4	3	3	5	5	5	34	Medium
Oxborrow et al. 2024 [36]	5	4	3	2	1	2	2	4	23	Low
Suresh et al. 2024 [37]	5	5	3	2	3	4	4	4	30	Medium

**Table 2:** Main themes and sub-themes emerging from qualitative analysis

Main Themes	Sub-theme	Category	Codes	References
Source of Carbon Emission	General Sources		Travel	[8, 19, 30]
			Building Energy	[8, 19]
			Electricity consumption	[7, 8]
			Procurement	[8, 19, 30]
			Waste	[2, 30]
	Contribution from dental care		Toothbrushes	[9]
			Examination	[19, 33]
			Radiographs	[19]
			Study Model	[19]
			Fluoride Varnish	[19, 34]
			Nitrous Oxide Sedation	[19, 31]
			Scale and Polish	[19]
			Amalgam Fillings	[19]
			Composite Restoration	[19]
			Glass Ionomer Fillings	[19]
			Endodontic Treatment	[7, 19]
			Dental crowns	[19]
			Extractions	[19]
			Denture-acrylic/metal	[19]
Strategies to promote sustainable dental practices	Clinical procedure related strategies	Reduce	Combine procedures- reduce appointments	[8, 19, 34]
			Reduce number of instruments in examination kit	[33]
			Reduce glove use	[1, 29]
			Reduce non-recyclable clinical waste	[2, 36]
			Reduce number of files in RCT/Single visit RCT wherever possible	[7]
			Remove Sterile packaging before starting treatment	[29]
		Reuse	Reusable bibs/gowns over disposable bibs/gowns	[7, 33]
			Reusable products	[29]
			Use reusable packaging that can be resterilised	[29]
		Alternative Materials	Environment friendly soap solutions	[7, 33]
			Environment friendly clothing bamboo fibre/recycled plastic	[33]
			Alternatives to iso-propyl alcohol	[7]
			Virgin pulp to sanitary paper to air hand drying	[7, 36]
		Treatment Strategies	Intra oral digital impressions	[1, 36]
			Treatment of preventable oral conditions	[32]
			Shift in endodontics from RCT to vital pulp treatment procedures	[7]
		Nitrous Oxide Management Strategies	Nitrous oxide scavenging machines	[19]
			Nitrous oxide neutralization/Crack Technology	[19, 31]
			Methoxyflurane as a substitute	[31]
	Dental Practice Management related strategies	Ground-level Strategies	Reviewing Expenditure	[19]
			Waste Segregation	[1, 2, 36]
			Collect and Recycle Stone	[33]
			Run dishwashers/washing machines when full	[33]
			Storage by digitization	[1, 30]
		Infrastructural Strategies	Energy saving-electric hand driers	[1, 37]
			LED sensors for lighting/LED Lighting	[1, 37]
			Solar Panels to power practice	[37]
			Rainwater collection tank	[35]
			Green Impact Toolkit recommendation	[35]
		Staff related strategies	Incentivize staff/patients to use low carbon transport options	[30]
			Increase environmental awareness and in-house sustainability initiatives	[1, 29, 37]
			Awareness on glove usage with clear guidelines	[29, 36]
			Action research	[1]
	Policy related strategies		Involve all stakeholders	[29, 37]
			Regulations to alter behaviour towards sustainability	[29]
			Subsidizing sustainable alternatives to disposable/single-use items	[36]
Challenges for sustainability			Infection control protocols	[1, 2, 29]
			Initial financial investment	[35]
Future research areas	Dental material related research		Surface Disinfectant alternatives	[33]
			Alternatives to single use items like stainless steel or biodegradable materials like bamboo	[7, 36]
			Biomimetic restorative solutions	[7]
			Environment friendly medicaments	[7]
	Research Methodology		Life cycle analysis	[8]
			Action Research	[1]

To provide a more in-depth understanding of the findings, qualitative thematic analysis was conducted on the selected articles, leading to the identification of four main themes concerning sustainable dental practices i.e., sources of carbon emissions, strategies to promote sustainable dental practices, challenges for sustainability, and future research areas. Table 2 depicts the detailed structured overview of these main themes, along with their associated sub-themes, categories, and codes.

The first main theme focused on sources of carbon emissions, which were broadly classified into two sub-themes: general and procedure specific contributors. This suggests that the environmental impact of dentistry encompasses both operational practices like travel, procurement, energy use and waste management as well as clinical care delivery including routine dental procedures and everyday items like toothbrushes, highlighting how even seemingly minor actions cumulatively contribute to the dentistry’s carbon footprint.

The second main theme presents a wide range of strategies to promote sustainable dental practices which emerged from the review. These were further grouped into three sub-themes based on their area of application: clinical procedure-related strategies, dental practice management related strategies, and policy related strategies. Clinical procedure related strategies focused on reducing emissions by reducing material use and minimizing unnecessary appointments, encouraging single-visit root canals, reusing bibs and gowns, and adoption of environmentally-friendly materials like bamboo and other biodegradable products. The integration of innovations such as digital impressions and a greater emphasis on preventive care were also highlighted. Attention was also drawn to nitrous oxide management, thereby reflecting on the importance of addressing often-overlooked but high-impact emission sources. Practice management strategies highlighted operational efficiency, waste segregation, record digitization, and sustainable infrastructure—such as solar panels and energy-efficient fixtures, indicating that sustainability must be built into everyday workflows, infrastructure, and procurement. Staff engagement was also identified as a critical component, underscoring that long-term change requires not only individual behaviour shifts but also cultural transformation, shared responsibility, and institutional support. Finally, policy-related strategies underscored the need for coordinated policy action to drive transformation within the profession. This includes developing clear regulatory frameworks, mandating sustainability practices, and fostering cross-sector collaboration among practitioners, manufacturers, administrators, educators, and policymakers to align healthcare delivery with environmental goals.

The third main theme highlighted several challenges in implementing sustainable dental practices, centring on the need to balance clinical safety, economic feasibility, and environmental responsibility. It was noted that while infection control protocols are essential for ensuring patient safety, they often conflict with sustainability goals, thereby underscoring the urgent need for innovations that maintain care standards without increasing environmental harm. Another significant obstacle identified was the financial limitation, notably the considerable upfront investment needed to transition to sustainable infrastructure.

Lastly, the fourth theme revolved around future research directions, emphasizing the need on material innovation to develop sustainable dental materials and medicaments, including biodegradable alternatives to single-use plastics such as bamboo, environmentally-friendly surface disinfectants and biomimetic restorative materials Additionally, the adoption of methodologically rigorous approaches such as life cycle analysis, along with qualitative techniques like action research to better understand behaviour and drive change, were highlighted as essential tools for generating robust, practice-relevant evidence to guide sustainable transformation in dental care.

## Discussion

This scoping review offers a comprehensive synthesis of 15 included studies shedding light on both the scale of the challenge and the opportunities for advancing sustainability in dental practice. Based on the CCAT scores, the majority of the included studies were rated as either high or medium quality, with only one study classified as low quality. This indicates that the overall evidence base is methodologically sound, allowing the findings of this review to be interpreted with a reasonable level of reliability. As detailed in the results section, four central themes were identified through thematic analysis: sources of carbon emissions, strategies to promote sustainability, barriers to implementation, and future research priorities. wide range of activities contributing to environmental impact, from clinical procedures and the use of everyday items like toothbrushes to travel, procurement, and waste management. A diverse set of strategies to integrate sustainability into dental practice emerged across clinical, managerial, and policy levels, emphasizing the need for material reduction, digital innovation, resource reuse, and stakeholder engagement. The challenges to sustainability in dentistry also surfaced which encompassed the conflict between infection control standards, patient safety, financial feasibility, and environmental responsibility. Lastly, the review highlights the need for continued research towards innovation in sustainable materials and adoption of appropriate research methodologies to drive meaningful, evidence-based transformation.

To guide a holistic interpretation, the following sections will explore these findings utilizing a recently proposed hierarchical framework for implementing sustainability in dentistry, examining potential interventions at the structural, practice, practitioner, and product levels to support meaningful and system-wide change.

### Structural level

This level of hierarchy addresses the existing socio-cultural and political context, as well as infection control guidelines. The policy strategies for sustainability in dentistry identified in this scoping review emphasize involving all stakeholders, including oral health practitioners, managers, practice owners, producers, suppliers, infection control experts, and waste management companies [29, 37]. Collaboration among these stakeholders is essential for fostering sustainable changes. Another crucial point is the impact of infection control policies and regulations on staff behaviour towards sustainability. Often, compliance with regulations and sustainable actions are in conflict, as recognized in a main theme emerging from this scoping review addressing challenges in achieving sustainability [1, 2, 29], thus emphasizing the necessity for clearer guidance [29]. Additionally, subsidizing private and public investments to develop sustainable alternatives to single-use and disposable items was suggested as a key measure at the structural level [36]. A qualitative exploration regarding sustainability in dental practices found that participants believed there was insufficient information in literature about sustainable dental practices and a lack of available options, even when they wished to adopt more sustainable methods and products [37]. A recent article addressed this concern by introducing an accessible and freely available carbon calculator. This tool aids in measuring and monitoring carbon emissions, thereby supporting dental professionals in their transition towards environmentally friendly practices [38].

### Dental practice level

This tier of the hierarchy focuses on the policies and practices implemented within an organization or a specific dental practice. The sub-theme derived from the qualitative data analysis that best encapsulates this level is the dental practice management strategies identified in this scoping review to promote sustainable dental practices, categorized into ground-level strategies, infrastructural strategies, and staff-related strategies.

Ground-level strategies encompass basic changes within the practice, such as transitioning to paperless operations with digitized storage [1, 30] and waste segregation [1, 2, 36]. Reviewing expenditure is a significant aspect within this category, as procurement has been identified as a major contributor to carbon emissions in dental practices [8, 19, 30]. Infrastructural strategies involve modifications within the dental clinic to promote water and energy-saving measures, as well as the use of renewable resources [1, 35, 37]. This is in line with the understanding that electricity usage [7, 8] is recognized as a significant contributor to carbon emissions in dental practices, emphasizing the importance of adopting energy-efficient practices moving forward [39]. A significant challenge in sustainability, particularly for infrastructural strategies, is the initial financial investment required to adopt new methods and make necessary changes [29].

Finally, staff-related strategies are crucial in raising awareness among staff members to reduce their carbon footprint and shift their mindset [1, 29, 36, 37]. This would likely partially address the resistance to change which often stems from lack of awareness, established habits, and workflow concerns, thereby posing a significant barrier to sustainable practices. Additionally, this requires institutional support as well as effective leadership endorsement to incorporate sustainability into dental practice. This intricate link between humans, resources, and the planet in the pursuit of sustainability, makes it a suitable area for conducting action research and placing participants at the center of decision-making as agents of change [40, 41]. Alongside initiatives such as engaging in action research [1], incentives can be provided for staff to use low-carbon transportation for commuting [30], considering that travel constitutes a significant source of carbon emissions in the field of dentistry [8, 19, 30]. Finally, integrating environmental literacy into dental curricula and continuing education can equip both emerging and practicing professionals with the knowledge and motivation needed to adopt sustainable practices, thereby laying a strong foundation for embedding a culture of sustainability within the dental profession [42].

### Oral health practitioner level

The sub-themes focusing on oral health practitioners primarily addressed identifying strategies at the clinical level to advance sustainability. However, it’s essential to recognize that sustainability in practice should encompass more than just reducing carbon footprint and should also prioritize delivering high-quality care within societal, economic, and environmental constraints. This introduces important ethical considerations in integrating sustainability into clinical decision-making. For instance, choosing tooth extraction solely because it has a lower carbon footprint than restorative options is not ethically sound. Similarly, single-visit root canal treatments should be performed only when clinically appropriate, not merely for the sake of reducing environmental impact. Moreover, practitioners must continually navigate the ethical dilemma between maintaining rigorous infection control standards—essential for patient safety—and implementing environmentally sustainable practices, often making decisions in ethically grey areas where clinical excellence and ecological responsibility must be carefully balanced.

In terms of sustainability, preventive measures such as fluoride varnish and fissure sealants are likely more sustainable because they reduce future dental care needs and associated carbon emissions [19, 43, 44]. Reduction has been recognized as crucial for minimizing environmental impact in the context of effective oral healthcare [39]. This approach can be integrated into high-quality oral care through the adoption of the four domains outlined by Martin and Mulligan: preventive care, operative care, integrated care, and responsibility for care [45]. This concept of reduction can be further implemented by minimizing patient travel indirectly through reducing the number of physical dental appointments, combining procedures [8, 19, 34], opting for single-visit root canal treatment whenever possible [7], coordinating family appointments, and utilizing digital technology such as remote clinical consultations and telemedicine [46–48].

Other strategies in clinical procedures aimed to utilize reusable and environmentally sustainable materials for commonly used items [7, 29, 33, 36], along with implementing measures to manage nitrous oxide within clinical environments [19, 31]. Nitrous oxide sedation also is an important contributor to the carbon footprint where this toxic greenhouse gas has a high global warming potential with one kilogram of nitrous oxide being equivalent to 298 kg of CO2 [19]. Proposals for managing nitrous oxide emissions in clinical settings include solutions such as nitrous oxide scavenging machines, cracking technology, and the use of methoxyflurane as an alternative [19, 31].

### Method or product level

This final, yet crucial, tier of hierarchy focuses on the methods and products employed in understanding the carbon footprint associated with dental care. By improving the understanding of how specific products and methods impact the environment, dental practitioners can identify patient-centered approaches that effectively minimize environmental impact. This focused primarily on understanding how dental care contributes to carbon emissions, encompassing factors such as toothbrushes, the nature and frequency of clinical procedures, the types of waste produced, and exploring environmentally-friendly alternatives to enhance sustainability. For instance, one study included in this review found that electric toothbrushes were significantly less sustainable compared to bamboo alternatives, impacting habitat, biodiversity, and land use 36 times more negatively [9]. Although an individual clinical examination tends to have a small carbon footprint, the sheer number and increased volume of these procedures contribute to the highest carbon footprint, followed by scale and polish [19]. The principles of reducing and reusing were applied at the method and product levels, advocating for a decrease in waste through the reduction of non-recyclable, single-use as well as paper items [2, 6, 28] and transitioning to intra-oral digital impressions [1, 36]. Additionally, promoting the reuse of products like gowns and bibs, and adopting reusable sterile packaging that can be sterilized multiple times [7, 29, 36], aligned with a move towards more environmentally friendly alternatives [7, 20].

### Future research areas

Replacing single-use items with more sustainable options such as biodegradable materials like bamboo or stainless steel can significantly reduce environmental impact. However, thorough research is needed to assess the safety implications of these alternatives before implementing them in clinical settings. Future research should also explore environmentally friendly medications such as naturally sourced irrigants antibacterial dressings as alternatives to cytotoxic standards, and biomimetic restorative solutions for minimally invasive regenerative endodontics [7, 33, 36]. Among the recommended research methodologies to better understand the environmental impacts of products and processes, life cycle impact assessment stands out as a key area for future research, which offers a globally accepted scientific and systematic approach to evaluate impacts from manufacturing through to disposal [8, 31].

While much research identifies different sources of carbon emissions related to dentistry, there is a noticeable absence of high-quality studies investigating the adoption of sustainable dental practices in the field. Such research could offer valuable insights into the challenges associated with promoting sustainability. Additionally, there is a need for more qualitative research globally to better understand the necessary changes and approaches needed to enhance sustainability in dental practices. More specifically, action research is another methodology that has emerged, helping to understand the reasons behind the decisions made by members of a dental team while also collaboratively generating ideas to engage in decision-making processes that affect them and their environment. This participatory approach is employed to implement joint interventions aimed at mitigating the environmental impact of dental practices and promoting sustainability [1].

### Limitations of the study

One primary limitation of this study was its reliance on searches from only four electronic databases, which may have resulted in the exclusion of relevant studies. This review excluded non-English and grey literature due to resource, time, and translation constraints, which may have limited the inclusion of relevant regional studies, particularly those published in local languages. While the focus on peer-reviewed sources ensured methodological rigor and data reliability, future reviews would benefit from incorporating multilingual and grey literature to provide a more comprehensive and globally representative synthesis.

## Conclusions

With an aim to systematically explore the existing literature, this scoping review synthesized findings from 15 eligible studies to provide a comprehensive overview of carbon emissions in dentistry and to explore pathways toward achieving environmental sustainability in dental practice. The qualitative thematic analysis helped to identify four main themes concerning sustainability in dentistry which included, sources of carbon emissions, strategies to promote sustainable dental practices, challenges in implementing sustainability and future research directions. These themes were organized and further discussed within a hierarchical framework encompassing four levels—structural, practice-based, practitioner-level, and material/method-based—offering a broader perspective on the multi-dimensional nature of sustainability in dentistry. Furthermore, this review highlights the need for future research to further investigate sustainable alternatives, conduct life cycle impact assessments, and undertake more qualitative studies to inform best practices. Overall, this review contributes to the growing discourse on sustainable healthcare by contextualizing dental practices within broader environmental goals. It provides a foundational evidence base to guide policy-makers, educators, and dental professionals in aligning oral healthcare delivery with environmental responsibility.

## Supplementary Information

Supplementary Material 1

Supplementary Material 2

Supplementary Material 3

Supplementary Material 4

## Data Availability

No datasets were generated or analysed during the current study.

## Abbreviations

CCAT: Crowe Critical Appraisal Tool
CO2: Carbon Dioxide
CO2eq: Carbon dioxide equivalents
ISO: International Organization for Standardization
PRISMA: Preferred Reporting Items for Systematic Reviews and Meta-Analyses
PRISMA: ScR-Preferred Reporting Items for Systematic Reviews and Meta-Analyses for Scoping Reviews
RCT: Root canal treatment
WHO: World Health Organization
