# Supplementary material for: A scoping review exploring carbon emissions in dentistry—a step towards sustainability
Source: BMC Oral Health. 2025 Oct 27;25:1674. doi: 10.1186/s12903-025-06952-w (PMC12557921; doi:10.1186/s12903-025-06952-w)
Supplement: Supplementary file 1 — Supplementary Material 1 [file 12903_2025_6952_MOESM1_ESM.docx]

| **Supplementary file 4 depicting data extraction table** | | | | | | | | | |
| --- | --- | --- | --- | --- | --- | --- | --- | --- | --- |
| **Title and Author(s) of Article** | **Purpose and Theoretical/Conceptual Orientation**: | **Research Questions and/or Hypotheses**: | **Sample Description** | **Methods and Analysis** | **Key Measures** | **Main Findings or Results:** | **Author(s)-stated limitations of study:** | **Strengths and importance of study** | **CCAT Score / Quality of the study** |
| Taking a bite out of Scotland’s dental carbon emissions  in the transition to a low carbon future.  Duane et al. 2012 | Greenhouse gas emissions are a major hazard to world health, the effort must be taken to minimize them. The first attempt to measure the carbon emissions of a national dental service is presented in this paper. | Not mentioned | Both direct and indirect emissions were included to determine total CO_2_ equivalent. | In order to determine carbon emission profiles, a process analysis (bottom-up) technique was used for direct emissions and a top- down technique for indirect emissions.  Bottom-up approach included building energy, travel, waste and water production.  Top-down approach included procurement | Important actions consist of constructing a process map, setting priorities, examining limits, gathering information and  figuring out the footprint. | 3.6% of Scotland's total carbon footprint and 23% of the country's public sector emissions, was the estimated total greenhouse gas footprint of NHS Scotland in 2004. The study's findings show that 4% of NHS Scotland's overall carbon footprint comes from the Community and Salaried Dental Service. | The input output analysis has limitations as dental expenses need to be categorised and there are no accurate data on dental product life. | Frequent "checkups" of carbon emissions in the dental industry, along with a deeper comprehension of the main carbon sources and related mitigation strategies, will yield more accurate data to support the difficult policy decisions surrounding the optimization of health benefits and the reduction of social, economic, and environmental drawbacks. | 26 /Medium |
| Exploring attitudes and  knowledge of climate change  and sustainability in a dental  practice: A feasibility study into  resource management. Grose et al. 2016 | To know about the behaviour and attitude of dental staff in resource management | To find out the level of awareness and knowledge regarding sustainable dentistry | All members of a mixed NHS / private dental practice in North- Devon were taken for interviews | It is a qualitative interview study | How the behaviour of staff could be made adoptable of methods to reduce, reuse and recycle in a dental setup. | As waste amount generated was more, the waste removal costs increased by 58%(predominantly by increased production).  The participants interviewed for this study expressed a range of attitudes from interest to denial and some participants avoided resource management due to lack of clear guidance. There were negative and positive responses. Some participants were aware of recycling of paper and cardboard but some had no idea about it. | The senior staff when questioned showed their confusion and blamed the guideline’s lack of clarity regarding waste management and infection control policies.  Major junior staff were worried with the issue of clinical waste storage as the space to store was not there. | Various recommendations for sustainability were given:  1. Purchasing methods to be reviewed .  2. Reduce packaging of sterile instruments  3. Reduce tissue and glove use  4. Discussion sessions among staff should be planned and safe working practices should be followed.  5. All items inside the treatment room should not be considered as clinical waste. | 33/Medium |
| What’s in a bin: A case study of  dental clinical waste composition  and potential greenhouse gas  emission savings.  Richardson et al. 2016 | The study's goal is to learn more about the biomedical waste produced by dentists, including its composition and management practices, in order to lower carbon emissions and make the dental field more environmentally friendly. | In addition to determining the amount and kind of clinical dental waste, the study attempted to determine whether it is feasible to calculate the possible cost and carbon savings associated with proper recycling and segregation. The study's main focus was on dental consumables' use and disposal. | An NHS and private dental practice in North Devon served as the study's site. During a predetermined number of therapy sessions, the research team audited the clinical waste generated in the clinic. | One dental practice's clinical waste was weighed and categorized using an auditing technique. Waste was gathered and examined twice in a row and two different time intervals. Waste was recorded and categorized based on the kind of material and how frequently it was discovered. The everyday objects were photographed. Every item was recognized, weighed, and the quantity of waste(frequency and mass) was noted. Two categories were created out of the clinical waste: recyclable waste and non-recyclable waste. | 1. The clinical waste audit was carried out twice, with a five-week gap between each session, on the days that were close to collection deadlines.  2. The use and disposal of dental consumables is the study's main topic. 3. Quality control over data entry has been ensured by:   A member of the audit team who was not involved in the audit | Items that are disposed of during clinic appointments were paper tissues,  gloves and the use of sterile wrapping  Paper tissues accounted for about 3000g of the 6,720g total waste, nitrile gloves for nearly 1,800g, and sterile wrapping for the remaining material. The most efficient way to separate sterile wrapping (for recycling) before it comes into touch with any contamination might potentially cut waste production at this practice by up to 5 kg per week. | The feasibility study shouldn't be generalized because it was only conducted in one dental practice.  Domestic waste and practice observations were not included in the waste audit. | The results helped the practitioner to understand the recognition of production and disposal of waste within the practice. | 37/High |
| An estimated carbon footprint of NHS primary dental  care within England. How can dentistry be more  environmentally sustainable?,  Duane et al. 2017 | The purpose of this study is to understand the association of  carbon emissions with commonly  performed dental procedures and to develop more sustainable  dental services. | Public Health England (PHE) commissioned a computation and analysis of the carbon footprint of important dental operations. (NHS) England wanted to improve sustainability of dental care. | The carbon emissions from personnel at primary care dental practices in NHS England were used to determine the carbon e missions of those practices. | The carbon emissions from patient travel, staff travel (including commuting and work-related travel), energy, water, and procurement were combined to determine the carbon emissions of primary care dental practices throughout NHS England. The data was gathered from April 2013 to March 2014. This article employed a process-based life cycle analysis | The NHS Business Service Authority (BSA) provided the data, Accounting data from NASAA, Information Services Division (ISD) Scotland, and recently released Scottish papers  were undertaken using a process-based and environmental input-output analysis. | An estimated 675 kilo-tonnes of greenhouse gas emissions were produced by NHS dentistry services in England measured in tonnes of carbon dioxide equivalents (tCO2e). Examinations contributed the highest proportion to this footprint followed by scale and polish and amalgam/composite restorations. From an emissions perspective, nearly 2/3 of emissions related to travel, 19% procurement and 15.3% related to energy use. | Regarding the distinction between the carbon emissions linked to the particular components of amalgam and composite fillings, no information is available. Due to the lack of precise material data, dentists are unable to confidently assess the long-term viability of various individual treatments.  . | Reducing carbon emissions is only one aspect of sustainability; another is providing high-quality healthcare while staying within legal, social, and environmental constraints. Also dental team must act with integrity, taking a holistic approach to patient care and adopting sustainable dental practices. | 25/Medium |
| Developing sustainability in a dental practice  through an action research approach,  Grose et al.  2018 | In a dental practice staff should become enivornmentally literate and know the climate change issues so this study is aimed at developing interventions to enhance sustainability at a single dental practice | Involving participants in an action research method and allowing them to reflect on the engagement process and subsequent practice changes | Action research was used in this mixed-methods study to create interventions, and both qualitative and quantitative data were included. | By taking notes and digitally capturing the group con versations, data was collected. Participants were provided with a reflective logsheet so they could offer feedback on any concerns with the sustainability options selected. Focus group data were subjected to thematic analysis. | In 2017, action research was conducted from January to July. A sequence of actions were taken to include people in decision-making and obtain feedback. A number of focus groups for action research were held, led by experienced qualitative researchers | Interventions aimed at enhancing sustainability within the practice were done.  Staff was made aware of the need to reduce the glove use. For energy saving, light sensors and low energy light bulbs were used. | This study was conducted over a 6 month period at a single dental practice so its results cannot be generalised to other contexts. The team's suggested actions did not address travel, which was found to be the main source of the carbon footprint associated with NHS dental services. | The study's primary goal of including employees in a transformation process to increase sustainability was accomplished. | 28/ Medium 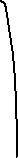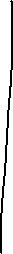 |
| Environmental sustainability in endodontics.  A life cycle assessment (LCA) of a root canal  treatment procedure,  Duane et al. 2020 | Purpose was to make endodontic operations environmentally sustainable and to evaluate the environmental impact of the RCT process by looking at its life cycle analysis. | The study was done to determine which elements of a standard RCT method have the greatest potential environmental impact and to evaluate and quantify the life cycle of an RCT. | The life cycle of a typical, two-visit RCT was assessed using a life cycle assessment (LCA) at faculty of dentistry Malmo University in Sweden.  The equipment and goods under analysis were those found in the standard kits provided to the faculty for treatment. | The functional unit for this investigation was defined as a single RCT procedure. The manufacturing, usage, sterilisation, disinfection, and disposal of all single-use and disposable instruments; the creation, cleaning, and discarding of dental garments; the water and energy consumption related to  the dentist's hand washing, the use and sanitization of the dental unit, and the sterilization and disinfection of the equipment were all covered. | One RCT technique served as the study's functional unit definition.  The production process, sterilization, disposal, and waste management of dental hygiene supplies, as well as energy and water consumption and dentist hand washing, were all considered inclusion criteria. Large equipment and the faculty building's construction were eliminated based on certain criteria. It also did not include travel for staff or patients. | The carbon dioxide equivalent emissions from an RCT operation are 4.9 kg.  One major factor in the reduction of ozone is dental clothing. The biggest causes of global warming include electricity, disposable bins, soap, detergent, surface disinfection, cardboard packaging, and single-use stainless steel instruments. | Since two sessions are the typical amount of time needed to complete an RCT, it is assumed that the procedure was finished in two sessions but the precise number of sessions required, however, depends on the dentist, patient-related factors, and tooth-related factors. As a result, it may take less or noticeably longer than two consultations to finish the treatment. | One potential method to enhance the environmental impact of an RCT is to employ minimally invasive regeneration endodontic techniques that aim to promote the repair or regeneration of damaged pulp tissue. | 37/ High |
| Combining evidence-based healthcare with  environmental sustainability: using the toothbrush  as a model,  Lyne et al. 2020 | Purpose of this study is to compare the sustainability of various toothbrush kinds and determining which life cycle elements have the greatest environmental impact | To find out which toothbrush type would be better for the patients and also environmentally sustainable. | Four types of toothbrush were taken for the study:  plastic manual, bamboo manual, plastic manual with replaceable head, electric. Each kind of toothbrush was represented by four distinct products available in the UK. | In collaboration with the Dublin Dental University Hospital, the Eastman Dental Hospital in London conducted a comparative life cycle assessment (LCA) of four distinct toothbrush types. | This study quantifies the environmental impact of the toothbrush—possibly the most widely used medical device globally—using the life cycle assessment (LCA) approach. | The results of the study indicated that the electric toothbrush had the biggest environmental impact. The manual toothbrushes with changeable plastic heads and those made of bamboo had the least amount of environmental impact in 11 and 5 of the impact categories, respectively. | Comparing various healthcare items using LCA has its limits. Interpreting the results might be challenging due to the variety of effect categories. | The outcomes of this study may have implications for toothbrush manufacturers, public health programs, oral health guidelines, and individual consumer choice. This LCA is beneficial for NHS and to guide healthcare policies and recommendations. | 29/ Medium |
| The life cycle analysis of a dental examination: Quantifying  the environmental burden of an examination in a hypothetical  dental practice,  Borglin et al. 2021 | To do LCA of a dental examination to know the environmental impact so that sustainable dentistry can be planned | This study will quantify the burden or impact on environment in a hypothetical dental practice | Oral examination of one patient in a hypothetical dental practice is performed. | LCA of dental examination to know the burden on environment by Ecoinvent version 3.5 database and LCA software tool open LCA version 1.10 | Major contributors to the environmental harm are identified in this study | Soaps, detergents, disposable bibs, clothing made of stainless steel, water use, waste water, and disposable bibs are the main sources of environmental damage caused by examination procedures. | So many assumptions and exclusions were made and there was bias in the origin and quantity of the products used. | The major contributors to environmental harm are identified in this study. Small changes in everyday practice can help to reduce environmental impact. | 36/ High |
| The environmental impact of community caries  prevention – part 1: fluoride varnish application, Lyne et al. 2022 | The aim is to quantify the impact on environment by fluoride application in dental practice | To compare the results of fluoride varnish application in schools and in a dental practice | An individual five year old child who is receiving FV application twice in an year | LCIA life cycle impact assessment of FV application twice in a year in a five year old child | FV is used as a community level caries preventive measure and it also has environmental impact | FV application during an existing dental appointment is best as it has the lowest environmental impact | In a single school visit, all children can not be available or reached.  FV given only to the children who regularly attend a dental practice will leave many children without FV application who do not generally get access to a dental clinic. | The assumptions that were made according to the LCA of environmental impact in this study are relevant and are applicable to most of the services in UK. | 34/ Medium |
| Facilitators and Barriers to Implementing  Sustainability in Oral Health Care,  Volgenant et al. 2022 | The purpose of this study is to find out facilitators and barriers in implementing sustainable practices in Dutch dental care | Understanding barriers and facilitators so that sustainable dentistry is practiced | 14 stake holders were interviewed which included  dentists, dental hygienists, and dental  assistants. Practice owners and managers,  distributors of  dental supplies and services were also identified as relevant  stakeholders. | Semi-structured interviews of the fourteen stake holders were done in this qualitative type of research study. | Facilitators and barriers are studied to implement sustainable dental practice | 1.At structural level, all of the participants agreed that infection control guidelines pose hindrances in the sustainability practices in oral health care. Example single use disposables like gloves and masks. And infection control guidelines do not recommend recycling.  2.All dental practice level, measures like LED lighting or providing sensors instead of light switches can bring down carbon emission due to energy use.  3.Female practitioner take more sustainable actions both at work and at home  4.As sustainable products are costly, the price acts as a barrier to sustainability | Limited knowledge and limited awareness are the hindrance in oral health care. | Facilitators of sustainable practices are the growing awareness to do something positive for man and his environment. | 32/ Medium |
| What is the environmental footprint of a dental  practice? A life cycle analysis (Part 1), Duane & Steinbach 2023 | A life cycle analysis of a dental clinic to calculate the environmental footprint | To know the changes of carbon footprint factors as compare to the original study. | A full time dental clinic operating 220 days/ year with one clinician and one dental nurse and half a receptionist was studied. | LCA of a full time dental clinic (220 days/year) to calculate carbon footprint | The environmental footprint of dental waste was higher than 2015 data. | Carbon emission are more by staff and patient travel and waste generated. Water use emissions are less comparatively. | A more detailed CFP (carbon footprint) analysis is needed so that carbon hotspots of dental procurement are found.  More information is required regarding the use of water in a community dental service. | This study has given an updated estimation of the carbon footprint of a dental practice | 33/ Medium |
| The environmental impact of nitrous oxide inhalation sedation  appointments and equipment used in dentistry,  Fennell-Wells et al. 2024 | Life cycle impact assessment (LCIA) of nitrous oxide use in a patient with dental appointment and to calculate carbon emission with comparison of single use and reusable equipment | --- | In a community dental service (CDS), one patient at a dental practice was studied. | LCIA of 30 minutes delivery of nitrogen: oxygen in 50:50 ratio as inhalation anaesthesia to one patient at a dental-practice and two types of equipment was compared that is reusable and single use equipment. | Clear documentation and transparency was maintained while doing LCIA as per ISO- 14040:2006 | In  NHS England out of the 2% contribution by anaesthetic gases to the carbon footprint, it is N_2_O which is responsible for 80% impact. The disposable (single use) equipment for N_2_O sedation causes significant larger environmental impact. | The difficulty in acquiring product manufacturing details makes this study unreliable. | LCIA is measured by an international standardised approach.  Clinicians service managers, health estate managers and policy makers can benefit from this study for improving patient care and reducing carbon footprint. | 38/ High |
| The environmental consequences of oral healthcare provision by the  dental team,  Martin et al. 2024 | The aim of this study is to do comparative ecological impact assessment of five patient categories from 6 years to 50 years to know total life time carbon footprint and single use plastic waste generation | The hypothesis states that good oral health care and prevention of preventable oral diseases has reduced environmental impact | Five patients representing different levels of dental disease between 6 year to 50 years for calculating carbon emissions and single use plastic waste generated. | Carbon emissions and SUP(single use plastic) waste calculations were done using current evidence based guidelines and published peer reviewed data. | The assessment of carbon emission and SUP is based on average restorative care levels of 50 year olds in UK | Low level of previous dental experience gives low carbon emissions and less number of single use plastic waste whereas very high level of previous dental experience results in increase number of single use plastic waste resulting in high impact on environment. | This study focuses on preventable oral conditions and has excluded developmental problems, orthodontic treatment and also elective procedures whereas all forms of oral health care puts impact on the environment | The findings of this study can be extrapolated to all regions on global basis  A good oral healthcare provision reduces number of patient travel and number of appointments with the help of telemedicine to reduce impact on the environment. | 34/ Medium |
| Simulation clinic waste audit assessment and  recommendations at the University of Washington School of  Dentistry,  Oxborrow et al. 2024 | The purpose of this study was to do waste audit at the University of Washington school of Dentistry and then provide waste reducing strategies at dental school setting in the United States | To form waste reduction strategies after conducting a waste audit study to make dentistry sustainable | 72 second year dental students performed restorative dentistry and 75 garbage bins were collected from the simulation clinic. Each category of waste was weighed. | A cross- sectional study where waste audit of waste bins was done and 72 second year students performed restorative dentistry. | A waste audit was conducted and reduction measures were recommended. | Plastic’ waste was the largest. Second largest category was the ‘paper’ and third largest is the impression material. | Recyclable items like cardboard and paper were found in the waste bins instead of recyclable material bins. Types and volume of waste generated during training exercises was more. | The suggestions given in this paper regarding implementing methods to reduce waste generation, and educating dental students about sustainable dentistry practices to decrease the impact on the environment. | 23/ Low 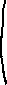 |
| A life cycle analysis of the environmental impact of  procurement, waste and water in the dental practice,  Suresh et al. 2024 | There is a global pressure towards a more sustainable provision of dental care so the green impact tool kit has been created and its recommendations are to be followed. | The study used LCA to quantitatively assess the possible benefits .of the actions recommended in the green impact tool kit under the theme of procurement, waste and water | Seven functional units were selected for the comparative LCA | A comparative life cycle assessment was conducted using Ecoinvent database 3.8 and were processed by open LCA v1.10.3 software | Various changes as per green tool kit are applied in the dental care practice to reduce carbon emissions. | The carbon footprint was significantly reduced after the recommendations were implemented. For example using water from a rainwater collection tank instead of the mains supply saved 30g CO_2_ eq. per patient, a 90% reduction in carbon footprint..  . | This study has limitations due to assumptions made and must be used only as guide. | This study provides evidence based guidance on changes that can be made to reduce CO_2_ eq. The actions recommended by green impact toolkit are easily implementable and are cost effective and can make dentistry sustainable. | 30/ Medium |
