# Supplementary material for: A scoping review exploring carbon emissions in dentistry—a step towards sustainability
Source: BMC Oral Health. 2025 Oct 27;25:1674. doi: 10.1186/s12903-025-06952-w (PMC12557921; doi:10.1186/s12903-025-06952-w)
Supplement: Supplementary file 2 — Supplementary Material 2 [file 12903_2025_6952_MOESM2_ESM.docx]

**Supplementary File 2**

**Search strategy across databases with keywords and Boolean operators**

| DATA BASE | SEARCH STRATEGY | RESULTS |
| --- | --- | --- |
| PUBMED | Search: **((dental) AND (dental care)) AND (dental waste)**  ("dental health services"[MeSH Terms] OR ("dental"[All Fields] AND "health"[All Fields] AND "services"[All Fields]) OR "dental health services"[All Fields] OR "dental"[All Fields] OR "dentally"[All Fields] OR "dentals"[All Fields]) AND ("dental care"[MeSH Terms] OR ("dental"[All Fields] AND "care"[All Fields]) OR "dental care"[All Fields]) AND ("dental waste"[MeSH Terms] OR ("dental"[All Fields] AND "waste"[All Fields]) OR "dental waste"[All Fields]) | 185 |
|  | **Search: ((((((dental) AND (dental care)) AND (dental waste)) AND (carbon)) AND (carbon footprint)) AND (emission)) AND (hospital) - Schema: all**  "dental"[All Fields] AND ("dental"[All Fields] AND "care"[All Fields]) AND ("dental"[All Fields] AND "waste"[All Fields]) AND "carbon"[All Fields] AND ("carbon"[All Fields] AND "footprint"[All Fields]) AND "emission"[All Fields] AND "hospital"[All Fields] | 0 |
|  | Search: **((((((dental) AND (dental care)) AND (dental waste)) AND (carbon)) AND (carbon footprint)) AND (emission)) AND (hospital)**  ("dental health services"[MeSH Terms] OR ("dental"[All Fields] AND "health"[All Fields] AND "services"[All Fields]) OR "dental health services"[All Fields] OR "dental"[All Fields] OR "dentally"[All Fields] OR "dentals"[All Fields]) AND ("dental care"[MeSH Terms] OR ("dental"[All Fields] AND "care"[All Fields]) OR "dental care"[All Fields]) AND ("dental waste"[MeSH Terms] OR ("dental"[All Fields] AND "waste"[All Fields]) OR "dental waste"[All Fields]) AND ("carbon"[MeSH Terms] OR "carbon"[All Fields] OR "carbons"[All Fields] OR "carbon s"[All Fields] OR "carbonates"[MeSH Terms] OR "carbonates"[All Fields] OR "carbonate"[All Fields] OR "carbonated"[All Fields] OR "carbonating"[All Fields] OR "carbonation"[All Fields] OR "carboneous"[All Fields] OR "carbonization"[All Fields] OR "carbonizations"[All Fields] OR "carbonize"[All Fields] OR "carbonized"[All Fields] OR "carbonizing"[All Fields] OR "carbonous"[All Fields] OR "fizzy"[All Fields]) AND ("carbon footprint"[MeSH Terms] OR ("carbon"[All Fields] AND "footprint"[All Fields]) OR "carbon footprint"[All Fields]) AND ("emission"[All Fields] OR "emission s"[All Fields] OR "emissions"[All Fields] OR "emissive"[All Fields]) AND ("hospital s"[All Fields] OR "hospitalisation"[All Fields] OR "hospitalization"[MeSH Terms] OR "hospitalization"[All Fields] OR "hospitalised"[All Fields] OR "hospitalising"[All Fields] OR "hospitality"[All Fields] OR "hospitalisations"[All Fields] OR "hospitalizations"[All Fields] OR "hospitalize"[All Fields] OR "hospitalized"[All | 0 |
|  | Fields] OR "hospitalizing"[All Fields] OR "hospitals"[MeSH Terms] OR "hospitals"[All Fields] OR "hospital"[All Fields]) |  |
|  | Search: **((dental waste) AND (carbon footprint)) AND (hospital)**  ("dental waste"[MeSH Terms] OR ("dental"[All Fields] AND "waste"[All Fields]) OR "dental waste"[All Fields]) AND ("carbon footprint"[MeSH Terms] OR ("carbon"[All Fields] AND "footprint"[All Fields]) OR "carbon footprint"[All Fields]) AND ("hospital s"[All Fields] OR "hospitalisation"[All Fields] OR "hospitalization"[MeSH Terms] OR "hospitalization"[All Fields] OR "hospitalised"[All Fields] OR "hospitalising"[All Fields] OR "hospitality"[All Fields] OR "hospitalisations"[All Fields] OR "hospitalizations"[All Fields] OR "hospitalize"[All Fields] OR "hospitalized"[All Fields] OR "hospitalizing"[All Fields] OR "hospitals"[MeSH Terms] OR "hospitals"[All Fields] OR "hospital"[All Fields]) | 7 |
|  | Search: **((dental care) AND (hospital waste)) AND (carbon emission) - Schema: all**  "dental"[All Fields] AND "care"[All Fields] AND ("hospital"[All Fields] AND "waste"[All Fields]) AND ("carbon"[All Fields] AND "emission"[All Fields]) | 0 |
|  | Search: **((dental care) AND (hospital waste)) AND (carbon emission)**  ("dental care"[MeSH Terms] OR ("dental"[All Fields] AND "care"[All Fields]) OR "dental care"[All Fields]) AND (("hospital s"[All Fields] OR "hospitalisation"[All Fields] OR "hospitalization"[MeSH Terms] OR "hospitalization"[All Fields] OR "hospitalised"[All Fields] OR "hospitalising"[All Fields] OR "hospitality"[All Fields] OR "hospitalisations"[All Fields] OR "hospitalizations"[All Fields] OR "hospitalize"[All Fields] OR "hospitalized"[All Fields] OR "hospitalizing"[All Fields] OR "hospitals"[MeSH Terms] OR "hospitals"[All Fields] OR "hospital"[All Fields]) AND ("waste"[All Fields] OR "waste s"[All Fields] OR "wasted"[All Fields] OR "wasteful"[All Fields] OR "wastes"[All Fields])) AND (("carbon"[MeSH Terms] OR "carbon"[All Fields] OR "carbons"[All Fields] OR "carbon s"[All Fields] OR "carbonates"[MeSH Terms] OR "carbonates"[All Fields] OR "carbonate"[All Fields] OR "carbonated"[All Fields] OR "carbonating"[All Fields] OR "carbonation"[All Fields] OR "carboneous"[All Fields] OR "carbonization"[All Fields] OR "carbonizations"[All Fields] OR "carbonize"[All Fields] OR "carbonized"[All Fields] OR "carbonizing"[All Fields] OR "carbonous"[All Fields] OR "fizzy"[All Fields]) AND ("emission"[All Fields] OR "emission s"[All Fields] OR "emissions"[All Fields] OR "emissive"[All Fields]) | 0 |
|  | Search: **((dental waste) AND (emission)) AND (hospital)**  ("dental waste"[MeSH Terms] OR ("dental"[All Fields] AND "waste"[All Fields]) OR "dental waste"[All Fields]) AND ("emission"[All Fields] OR "emission s"[All Fields] OR "emissions"[All Fields] OR "emissive"[All Fields]) AND ("hospital s"[All Fields] OR "hospitalisation"[All Fields] OR "hospitalization"[MeSH Terms]  OR "hospitalization"[All Fields] OR "hospitalised"[All Fields] OR "hospitalising"[All Fields] OR "hospitality"[All Fields] OR "hospitalisations"[All Fields] OR "hospitalizations"[All Fields] OR "hospitalize"[All Fields] OR "hospitalized"[All Fields] OR "hospitalizing"[All Fields] OR "hospitals"[MeSH Terms] OR "hospitals"[All Fields] OR "hospital"[All Fields]) | 25 |
| SCOPUS | TITLE-ABS-KEY ( dental AND waste ) AND TITLE-ABS-KEY ( carbon AND emission ) ) | 22 |
| WEB OF SCIENCE | ((ALL=(dental care )) AND ALL=(dental waste)) AND ALL=(carbon emission) | 9 |
|  | ((ALL=(dental waste)) AND ALL=(carbon emission)) AND ALL=(dental hospitals) | 3 |
| EMBASE | (**'dental care'**/exp OR **'dental care'** OR ((**'dental'**/exp OR **dental**) AND (**'care'**/exp OR **care**))) AND (**'dental waste'**/exp OR **'dental waste'** OR ((**'dental'**/exp OR **dental**) AND (**'waste'**/exp OR **waste**))) AND (**'carbon emission'**/exp OR **'carbon emission'** OR ((**'carbon'**/exp OR **carbon**) AND (**'emission'**/exp OR **emission**))) | 5 |
|  | (**'dental waste'**/exp OR **'dental waste'** OR ((**'dental'**/exp OR **dental**) AND (**'waste'**/exp OR **waste**))) AND (**'carbon emission'**/exp OR **'carbon emission'** OR ((**'carbon'**/exp OR **carbon**) AND (**'emission'**/exp OR **emission**))) AND (**'dental hospital'** OR ((**'dental'**/exp OR **dental**) AND (**'hospital'**/exp OR **hospital**))) | 11 |
